# An epigenetic signature of adhesion molecules predicts poor prognosis of ovarian cancer patients

**DOI:** 10.18632/oncotarget.18515

**Published:** 2017-06-16

**Authors:** Ping-Ying Chang, Yu-Ping Liao, Hui-Chen Wang, Yu-Chih Chen, Rui-Lan Huang, Yu-Chi Wang, Chiou-Chung Yuan, Hung-Cheng Lai

**Affiliations:** ^1^ Graduate Institute of Medical Sciences, National Defense Medical Center, Taipei, Republic of China; ^2^ Division of Hematology & Oncology, Department of Internal Medicine, Tri-Service General Hospital, National Defense Medical Center, Taipei, Republic of China; ^3^ Department of Obstetrics and Gynecology, School of Medicine, College of Medicine, Taipei Medical University, Taipei, Republic of China; ^4^ Division of Research and Analysis, Food and Drug Administration, Ministry of Health and Welfare, Taipei, Republic of China; ^5^ Department of Obstetrics and Gynecology, Shuang Ho Hospital, Taipei Medical University, New Taipei City, Republic of China; ^6^ Department of Obstetrics and Gynecology, Tri-Service General Hospital, National Defense Medical Center, Taipei, Republic of China; ^7^ Translational Epigenetic Center, Shuang Ho Hospital, Taipei Medical University, New Taipei City, Republic of China; ^8^ Department of Clinical Pharmacology, Xiangya Hospital, Central South University, Changsha, P. R. China; ^9^ Institute of Clinical Pharmacology, Central South University, Hunan Key Laboratory of Pharmacogenetics, Changsha, P. R. China

**Keywords:** ovarian cancer, DNA methylation, adhesion, prognosis

## Abstract

DNA methylation is a promising biomarker for cancer. The epigenetic effects of cell adhesion molecules may affect the therapeutic outcome and the present study examined their effects on survival in ovarian cancer. We integrated methylomics and genomics datasets in The Cancer Genome Atlas (n = 391) and identified 106 highly methylated adhesion-related genes in ovarian cancer tissues. Univariate analysis revealed the methylation status of eight genes related to progression-free survival. In multivariate Cox regression analysis, four highly methylated genes (*CD97*, *CTNNA1*, *DLC1*, *HAPLN2*) and three genes (*LAMA4*, *LPP*, *MFAP4*) with low methylation were significantly associated with poor progression-free survival. Low methylation of *VTN* was an independent poor prognostic factor for overall survival after adjustment for age and stage. Patients who carried any two of *CTNNA1*, *DLC1* or *MFAP4* were significantly associated with poor progression-free survival (hazard ratio: 1.59; 95% confidence interval: 1.23, 2.05). This prognostic methylation signature was validated in a methylomics dataset generated in our lab (n = 37, hazard ratio: 16.64; 95% confidence interval: 2.68, 103.14) and in another from the Australian Ovarian Cancer Study (n = 91, hazard ratio: 2.43; 95% confidence interval: 1.11, 5.36). Epigenetics of cell adhesion molecules is related to ovarian cancer prognosis. A more comprehensive methylomics of cell adhesion molecules is needed and may advance personalized treatment with adhesion molecule-related drugs.

## INTRODUCTION

Among the gynecological cancers, ovarian cancer is the leading cause of death globally [1, 2]. An estimated 75% of patients with ovarian carcinoma present with advanced disease [3]. Despite aggressive treatment with cytoreductive surgery and chemotherapy regimens, recurrence with intraperitoneal metastasis and chemoresistance are common. The overall cure rate of ovarian cancer patients is approximately 30% [4]. Tumor stage, residual tumor after surgery, histology, grade, and age are important prognostic factors for ovarian cancer [5]. However, ovarian cancer has complex biology and patients have diverse outcomes, even with the same risk factors and the same treatment. Currently, surgical stages, histological grades, and optimal debulking remain the major prognostic factors, which are also the main factors considered in the current treatment guidelines [6]. However, with increasing understanding of the molecular heterogeneity of ovarian cancers, a better prognostic biomarker for patient stratification and personalized treatment is needed. New prognostic biomarkers of ovarian cancer are needed.

Ovarian cancer cells detach from the primary tumor, and then disseminate in the peritoneal cavity and attach to the omentum and peritoneum as the initial steps of metastasis. Cell adhesion molecules (CAMs) are involved in all phases of cancer progression [7]. CAMs are classically categorized into four groups: the cadherins, the integrins, the selectins, and the immunoglobulin-like CAMs (Ig-CAMs) [8]. Different kinds of CAMs are essential for cancer cells to maintain their survival and to alter their local microenvironment so that it is more feasible to sustain and promote tumor development [8, 9]. CAMs are key players in the progression of ovarian cancer [10]. Reduced E-cadherin and β-catenin phenotypes are associated with advanced stage tumors, serous carcinomas, peritoneal metastasis, and larger residual tumor in ovarian cancer patients [11]. Loss of E-cadherin expression is associated with an unfavorable outcome [12, 13]. Loss of β3 integrin expression is a poor prognostic marker in ovarian cancer [14]. However, single integrins such as αvβ3 or α5β1 inhibitors fail to gain significant benefits in metastatic ovarian cancer [15, 16]. Epithelial cell adhesion molecule (EpCAM) is a member of the Ig-CAMs. The expression of EpCAM on the surface of normal ovarian epithelium is very low but is increased in ovarian cancer with different histology [17]. The prognosis as a result of expression of EpCAM in ovarian cancer is not clear [17, 18]. Anti-EpCAM monoclonal antibody (Catumaxomab) is useful for management of ovarian cancer with recurrent malignant ascites and can improve the quality of life (puncture-free time, time to next puncture) [19]. Although CAMs are critical in the progression of ovarian cancer, currently available treatments directed at these molecules do not improve clinical outcome. The reasons for the poor response to CAM-related therapies remain unknown.

In addition to genetics, epigenetics is also a driving force in cancer. Hypermethylation of tumor suppressor promoters and global hypomethylation are common during cancer development and progression [20]. DNA methylation markers are clinically useful for diagnostics, prognostics, and prediction of treatment response in cancer [21]. For instance, *O^6^-methylguanine DNA methyltransferase* (*MGMT*) methylation is a useful predictor of the responsiveness of tumors to alkylating agents, and survival of patients with glioma [22]. But there is no such methylation biomarker in ovarian cancer. Epigenetic alterations at specific CpG sites are associated with progression-free survival (PFS) and overall survival (OS) in patients with ovarian cancer [23–27]. Methylation of CAM may be correlated with risk and prognosis of ovarian cancer. High methylation of *cadherin 1* promoters is a potential biomarker for prediction of ovarian cancer risk [28]. High methylation of *intercellular adhesion molecule-1* and *opioid binding protein/cell adhesion molecule-like gene* promoter is associated with poor OS in patients with ovarian cancer [29, 30]. There is a need for a more comprehensive CAM methylomics study to stratify ovarian cancer patients for novel therapies.

Most previous studies have used a candidate-gene approach, and the selection of these genes is based on limited knowledge of the cancer biology. Moreover, genes with a similar function were not simultaneously included in analyses, and this may bias results. The present study integrates methylomics and genomics to explore the adhesion methylomics associated with survival in ovarian cancer.

## RESULTS

### Identification of adhesion-related genes with high methylation and low expression in ovarian cancer

**Figure 1:**
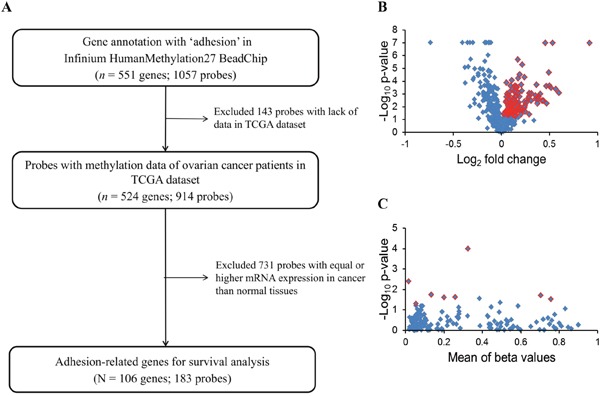
Flowchart of the present study **(A)** Adhesion-related genes from the Infinium HumanMethylation27 array were selected to integrate the GeneChip HT Human Genome U133 array data to select candidate genes with an mRNA expression significantly lower in malignant than in normal samples (*p* < 0.05) for survival analysis. **(B)** Selection of genes differentially expressed in normal and malignant samples from an expression microarray. Blue dots indicate 428 adhesion-related genes. The expression of 106 genes shown as red dots was significantly different between normal and malignant tissues (*p* < 0.05 by *t*-test). **(C)** There was a significant association of eight genes in TCGA ovarian cancer patients with PFS by a log-rank test (red dots) of 183 probes (blue dots).

Genes annotated as coding for adhesion in methylation profiles including 524 genes (914 probes) were selected. To narrow down the potential genes, we integrated the gene expression data, which include the normal ovarian and ovarian cancer samples. Genes with lower mRNA expression in cancer than in normal tissues were selected for survival analysis. There were 106 genes (183 probes) fulfilling these criteria. The selection flowchart is shown in Figure 1.

### Association between adhesion methylomics and prognosis

**Figure 2:**
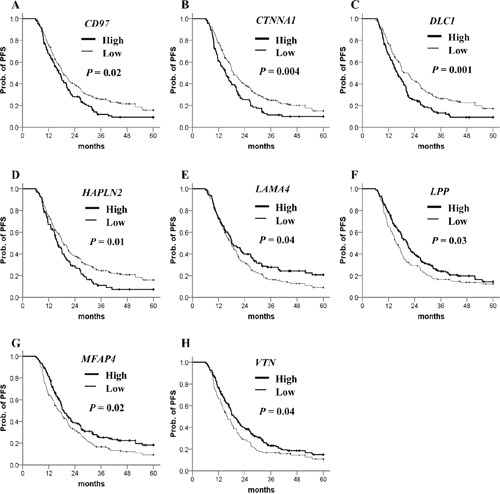
Kaplan–Meier plots for PFS analysis of candidate gene methylation in TCGA ovarian cancer dataset **(A–H)** PFS stratified by the methylation status of each candidate gene. Straight line: low methylation; bold line: high methylation. *P* values were calculated using a log-rank test.

**Figure 3:**
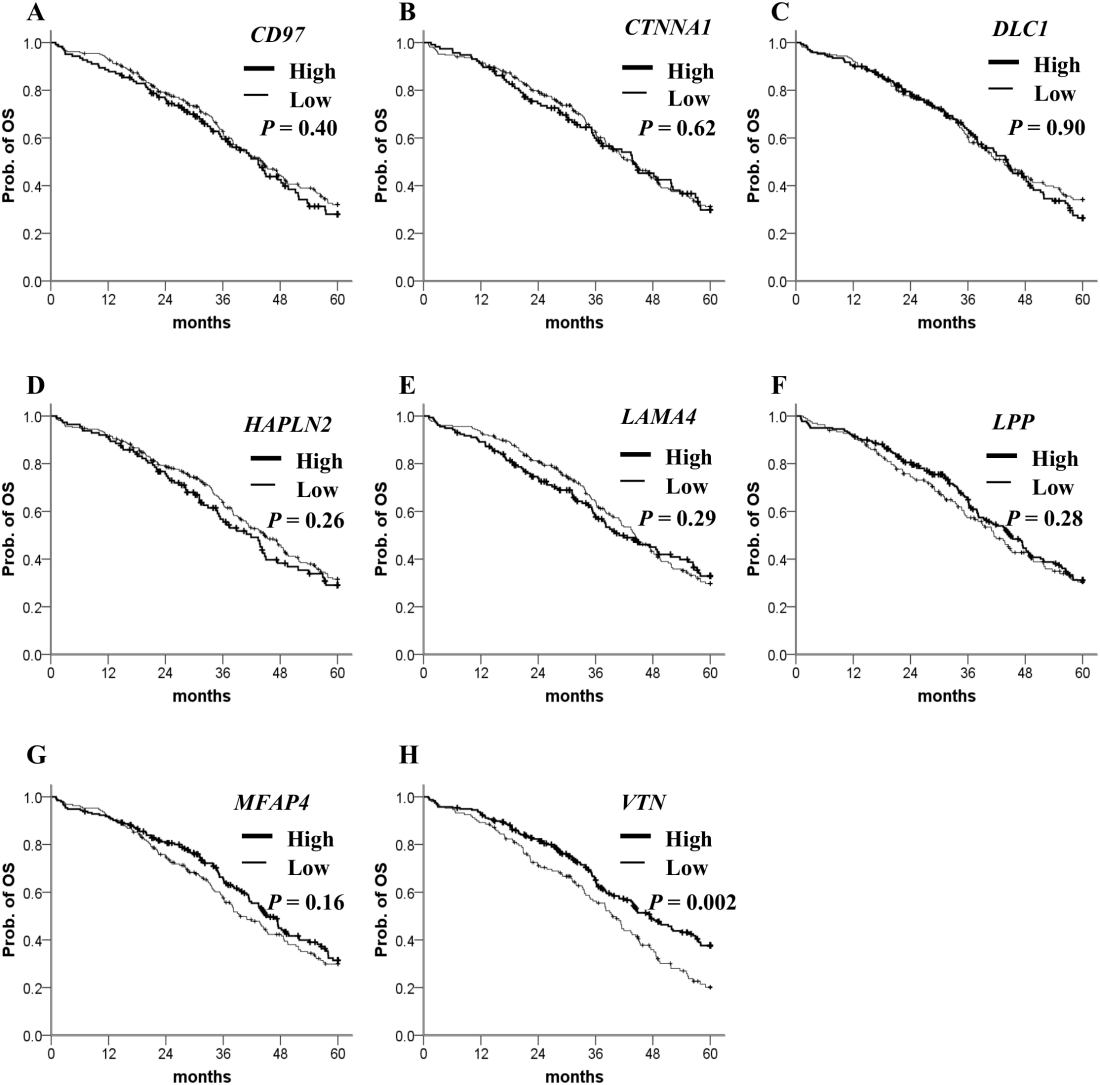
Kaplan–Meier plots for OS analysis of candidate gene methylation in TCGA ovarian cancer dataset **(A–H)** OS stratified by the methylation status of each candidate gene. Straight line: low methylation; bold line: high methylation. *P* values were calculated using a Breslow test.

To evaluate their clinical relevance, we analyzed the prognostic significance of methylation status of 106 genes using a dataset from The Cancer Genomic Atlas (TCGA). The patient clinicopathological features are presented in Table 1. Most of the ovarian cancer patients at diagnosis were at stage III/IV and high grade. The β-value distribution of candidate genes is presented in Table 2. By Kaplan–Meier analysis, patients with highly methylated *CD97*, *CTNNA1*, *DLC1*, *HAPLN2* and low methylation of *LAMA4*, *LPP*, *MFAP4*, *VTN* showed a significantly worse prognosis (Figure 2). In multivariate Cox regression analysis, FIGO stage was significantly correlated with PFS (Table 3). After adjusting for the stage, four highly methylated genes (*CD97*, *CTNNA1*, *DLC1*, *HAPLN2*) and three genes (*LAMA4*, *LPP*, *MFAP4*) with low methylation were found significantly associated with poor PFS (Table 3). In the Kaplan–Meier analysis for OS, only low methylation of *VTN* was significant for poor OS (Figure 3). Low methylation of *VTN* was an independent prognostic factor for OS after adjusting for age and stage (Table 4).

**Table 1:** Characteristics and clinicopathological features of TCGA ovarian cancer dataset

Characteristics		Median	Range
Age, years (*n* = 391)		59.6	30.5–87.5
		No. of patients	(%)^a^
FIGO stage	II	22	(5.6)
	III	307	(78.5)
	IV	62	(15.9)
Grade	G2	57	(14.6)
	G3	334	(85.4)
Platinum response^a^	Sensitive	193	(68.4)
	Resistant	89	(31.6)

FIGO: International Federation of Gynecology and Obstetrics. ^a^Percentages for platinum response were based on 282 tumor samples.

**Table 2:** The methylation level of adhesion-related candidate genes in TCGA ovarian cancer dataset

Gene name	β-Value	
	Median	Range
*CD97* (*n* = 391)	0.05	(0.02–0.18)
*CTNNA1* (*n* = 391)	0.01	(0.001–0.09)
*DLC1* (*n* = 382)	0.25	(0.03–0.88)
*HAPLN2* (*n* = 391)	0.11	(0.04–0.82)
*LAMA4* (*n* = 391)	0.18	(0.04–0.63)
*LPP* (*n* = 383)	0.78	(0.23–0.95)
*MFAP4* (*n* = 390)	0.39	(0.05–0.78)
*VTN* (*n* = 391)	0.74	(0.15–0.89)

**Table 3:** Univariate and multivariate Cox regression analysis of PFS of TCGA patients with high-grade serous ovarian cancer

Variable	Crude HR(95% CI)	*p*	Adjusted HR(95% CI)	*p*
Age (years)	1.00 (0.99, 1.01)	0.75	-	-
FIGO stage (stage III, IV vs II)	2.04 (1.14, 3.64)	0.02*	1.87 (1.04, 3.34)^b^	0.04*
Grade (G3 vs G2)	1.29 (0.91, 1.82)	0.15	-	-
Methylation status (low vs high)^a^				
*CD97*	0.74 (0.58, 0.95)	0.02*	0.76 (0.59, 0.98)	0.03*
*CTNNA1*	0.68 (0.52, 0.89)	0.005*	0.71 (0.54, 0.92)	0.01*
*DLC1*	0.66 (0.51, 0.85)	0.001*	0.69 (0.53, 0.88)	0.003*
*HAPLN2*	0.71 (0.54, 0.93)	0.01*	0.71 (0.55, 0.94)	0.01*
*LAMA4*	1.31 (1.01, 1.69)	0.04*	1.32 (1.02, 1.70)	0.04*
*LPP*	1.32 (1.03, 1.69)	0.03*	1.30 (1.01, 1.66)	0.04*
*MFAP4*	1.36 (1.06, 1.74)	0.02*	1.34 (1.05, 1.72)	0.02*
*VTN*	1.29 (1.01, 1.66)	0.05*	1.24 (0.96, 1.60)	0.09

HR: hazard ratio; CI: confidence interval. ^a^The HR adjusted by stage and gene methylation status. ^b^ The HR adjusted by *DLC1* gene methylation status. * Significantly correlated with outcome, *p* < 0.05.

**Table 4:** Univariate and multivariate Cox regression analysis of OS in TCGA patients with ovarian cancer

	Crude HR(95% CI)	*p*	Adjusted HR(95% CI)^a^	*p*
Age	1.02 (1.01, 1.04)	< 0.001*	1.02 (1.01, 1.04)	<0.001
Grade	1.47 (0.99, 2.19)	0.06	1.38 (0.92, 2.06)	0.12
FIGO stage				
Stage III, IV vs II	2.77 (1.23, 6.24)	0.01*	2.49 (1.10, 5.62)	0.03*
*VTN* methylation				
Low vs. High	1.52 (1.17, 1.99)	0.002*	1.49 (1.15, 1.95)	0.003*

^a^The HR adjusted by age, stage and methylation status. *p < 0.05.

### Prediction of PFS with adhesion methylomics signature in ovarian cancer

**Figure 4:**
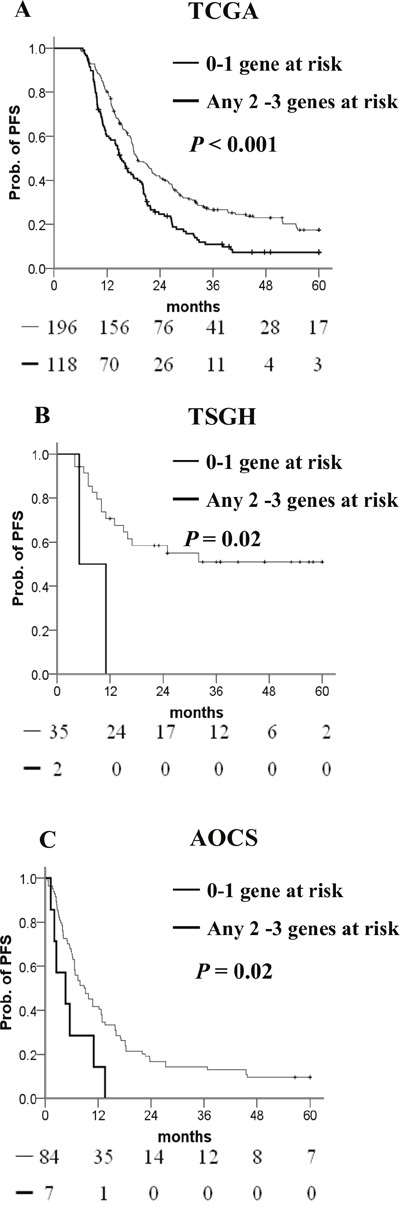
Kaplan–Meier curves of the risk groups **(A)** TCGA, **(B)** TSGH, and **(C)** AOCS ovarian cancer patients with the probability of PFS predicted by the *CTNNA1/DLC1/MFAP4* methylation signatures. PFS was stratified by the number of risk-related genes. Risk-related genes were defined as high methylation of *CTNNA1* or *DLC1*, and low methylation of *MFAP4*. Straight line: 0–1 risk-related genes; bold line: any 2–3 risk-related genes. *P* values were calculated using a log-rank test.

We used a stepwise Cox proportional hazards model to generate a combination of adhesion methylomics for better prognostic prediction, which resulted in a 3-gene signature (*CTNNA1*, *DLC1*, *MFAP4*). High methylation of *CTNNA1* and *DLC1* and low methylation of *MFAP4* indicate high risk. Patients carrying three or any two of these genes had a greater risk of shorter PFS than those with none or only one risk-related gene in the Kaplan–Meier analysis (Figure 4A). Multivariate Cox regression analysis confirmed that possessing three or any two risk-related genes was an independent risk factor for PFS when compared with those possessing none or any one risk-related gene (Table 5). When we looked at the platinum response of ovarian cancer in TCGA dataset, patients carrying three or any two risk-related genes had a significantly poorer PFS in the platinum-sensitive group. This difference was not statistically significant in the platinum-resistant group. The drug response and adhesion molecule status warrants further investigations. (Supplementary Figure 1A-1C).

**Table 5:** Univariate and multivariate Cox regression analysis of PFS of patients in TCGA, TSGH, and AOCS groups with high-grade serous ovarian cancer

Variable	Crude HR (95% CI)	*p*	Adjusted HR^a^ (95% CI)	*p*
TCGA				
Age (years)	0.99 (0.98, 1.01)	0.75	-	-
FIGO stage (III, IV vs II)	2.04 (1.14, 3.64)	0.02*	1.88 (1.05, 3.36)	0.002*
Grade (G3 vs G2)	1.32 (0.93, 1.87)	0.12	-	-
Methylation signature				
* CTNNA1*, *DLC1*, *MFAP4*				
0–1 gene at risk	1.00 (ref.)		1.00 (ref.)	
Any 2–3 genes at risk	1.64 (1.27, 2.11)	<0.001*	1.59 (1.23, 2.05)	<0.001*
TSGH				
Age (years)	0.98 (0.94, 1.02)	0.29	-	-
FIGO stage (III, IV vs I, II)	8.69 (1.98, 38.13)	0.004*	13.06 (2.64, 64.72)	0.002*
Grade (G3 vs G2)	1.44 (0.57, 3.67)	0.15	-	-
Methylation signature				
* CTNNA1*, *DLC1*, *MFAP4*				
0–1 gene at risk	1.00 (ref.)		1.00 (ref.)	
Any 2–3 genes at risk	4.90 (1.07, 22.49)	0.04*	16.64 (2.68, 103.14)	0.003*
AOCS				
Age (years)	1.03 (0.99, 1.05)	0.08	-	-
FIGO stage (IV vs III)	0.61 (0.34, 1.10)	0.10	-	-
Grade (G3 vs G2)	0.90 (0.46, 1.74)	0.75	-	-
Methylation signature				
* CTNNA1*, *DLC1*, *MFAP4*				
0–1 gene at risk	1.00 (ref.)			
Any 2–3 genes at risk	2.43 (1.11, 5.36)	0.03*	-	-

HR: hazard ratio; CI: confidence interval. ^a^The HR adjusted by stage and methylation signature. * Significantly correlated with outcome, *p* < 0.05.

**Figure 5:**
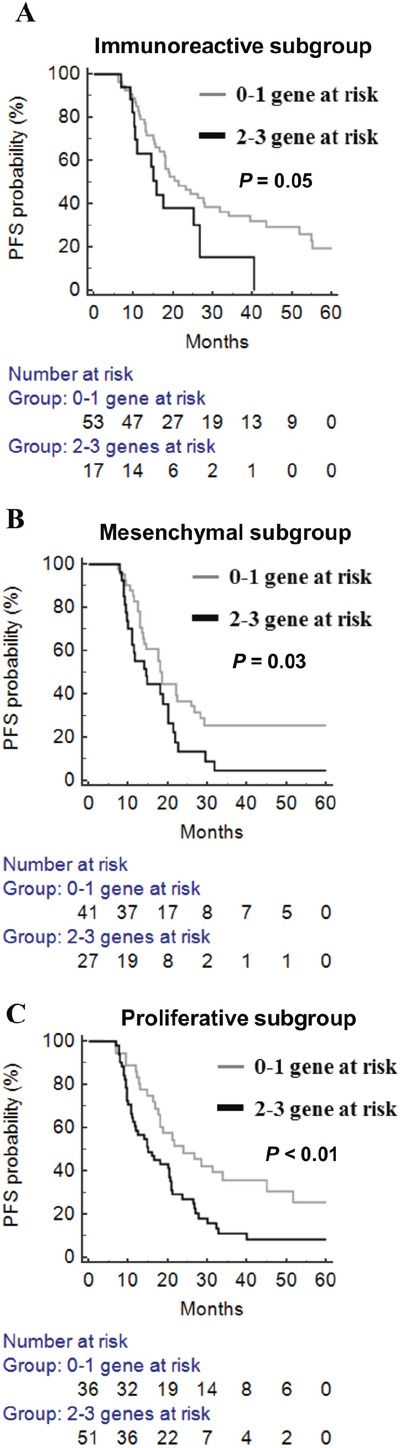
The prognostic significance of epigenetic adhesion signatures in different molecular subgroups in TCGA patients with high-grade serous ovarian cancer **(A)** Immunoreactive subgroup. **(B)** Mesenchymal subgroup. **(C)** Proliferative subgroup. Gray line: 0–1 risk-related genes; black line: any 2–3 risk-related genes.

The molecular background of ovarian cancer is heterogeneous. From the TCGA and AOCS datasets on high-grade serous ovarian cancers, four different molecular subtypes with distinct biology were identified [31, 32]. It is interesting to know whether this epigenetic adhesion signature is related to specific molecular subgroups. We examined the association between molecular subtypes and the epigenetic adhesion signature using the TCGA dataset. Indeed, there was a significant correlation between molecular subtypes and epigenetic adhesion signatures. Patients with three or any two risk-related genes were more common in the proliferative subtype (Table 6). In addition, the epigenetic adhesion signature was also correlated with PFS in immunoreactive, mesenchymal, and proliferative but not differentiated tumor subgroups (Figure 5A–5C).

**Table 6:** Correlation between epigenetic adhesion signatures and molecular subtypes in TCGA patients with high-grade serous ovarian cancer

Epigenetic Adhesion Signature			
Subtypes by gene expression	0-1 gene at risk	2-3 gene at risk	Chi-squared *P* value
Differentiated	66 (74%)	23 (26%)	<0.001*
Immunoreactive	53 (76%)	17 (24%)	
Mesenchymal	41 (60%)	27 (40%)	
Proliferative	36 (41%)	51 (59%)	

*Significantly correlated with outcome, p < 0.05.

### Independent validation of the adhesion methylomics signature

To validate the prognostic value of this signature, we generated 37 methylomics of ovarian cancer in our hospital. Patients carrying three or any two risk-related genes had a noticeably poorer PFS than those with none or any one risk-related gene (Figure 4B) in the Kaplan–Meier analysis. The signature was also validated in the AOCS dataset containing 91 patients with 450K methylomics. As expected, the 3-gene methylation signature showed a significant difference between 0–1 risk-related gene and any 2–3 risk-related genes (Figure 4C) in the AOCS dataset. The Cox proportional hazard model showed adjusted hazard ratio (HR)s for PFS of patients with 2–3 risk-related genes versus 0–1 risk-related genes in databases belonging to the TSGH and AOCS (Table 5).

### Integration of DLC1 methylation and FAK expression as a prognostic factor

**Figure 6:**
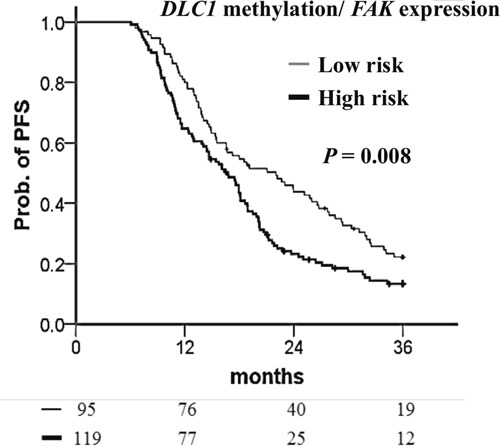
Kaplan–Meier curves of the risk groups for TCGA ovarian cancer patients with the probability of PFS predicted by combined *DLC1* methylation and *FAK* expression signatures PFS stratified by *DLC1* methylation with *FAK* expression signatures. Low-risk means *DLC1* low methylation with *FAK* low expression. The high-risk group included any combination of *DLC1* methylation with *FAK* expression other than the low-risk group. Straight line: low-risk; bold line: high-risk. *P* values were calculated using a log-rank test.

Focal adhesion kinase (FAK) regulates various intracellular signaling pathways and is associated with tumor progression [33]. In most ovarian cancers, *FAK* expression is aberrantly upregulated [34]. DLC1 has been reported to dephosphorylate FAK and inhibit the proliferation and migration of hepatocellular carcinoma cell lines [35]. Moreover, low expression of *DLC1* with high expression of pFAK Y397 (phosphorylated FAK) was detected in advanced ovarian cancers [36]. Therefore, we were interested in the combined effects of these two genes in ovarian cancers. In the present study, patients with highly methylated *DLC1* had shorter PFS than those with low methylation (Table 7). Patients could be stratified into two groups based on the combination of *DLC1* methylation and *FAK* expression. *DLC1* low methylation with *FAK* low expression indicates low risk. The high-risk group included any combination of *DLC1* methylation with *FAK* expression other than the low-risk group. The low-risk patients had a better 36-month prognosis (Figure 6). The median PFS of patients with low-risk was 5.3 months longer than that for patients in the high-risk group. Multivariate Cox regression analyses indicated the high-risk group had 1.42-fold the recurrence of the low-risk group and had an independent factor for PFS (Table 7).

**Table 7:** Univariate and multivariate Cox regression analysis of 3-years PFS of TCGA patients with ovarian cancer

	Crude HR(95% CI)	*p*	Adjusted HR(95% CI)	*p*
Age	1.01 (0.99, 1.02)	0.28		
Grade	1.45 (0.94, 2.23)	0.10		
FIGO stage				
Stage III, IV vs II	2.84 (1.25, 6.41)	0.01*	2.55 (1.12, 5.80)^b^	0.03*
*DLC1* methylation				
High vs low	1.49 (1.10, 2.01)	0.01*	1.39 (1.02, 1.88)^c^	0.04*
*FAK* expression				
High vs. low	1.15 (0.79, 1.69)	0.46	-	-
*DLC1*^m^ and *FAK*^e^				
High risk vs low risk^a^	1.51 (1.11, 2.05)	0.008	1.42 (1.04, 1.92)^c^	0.03*

^a^Low risk means *DLC1* low methylation and *FAK* low expression. The high-risk group included any combination of *DLC1* methylation with *FAK* expression other than the low-risk group. ^b^The HR adjusted by *DLC1*^m^ and *FAK*^e^ signature. ^c^The HRs adjusted by stage. *p < 0.05.

**Figure 7:**
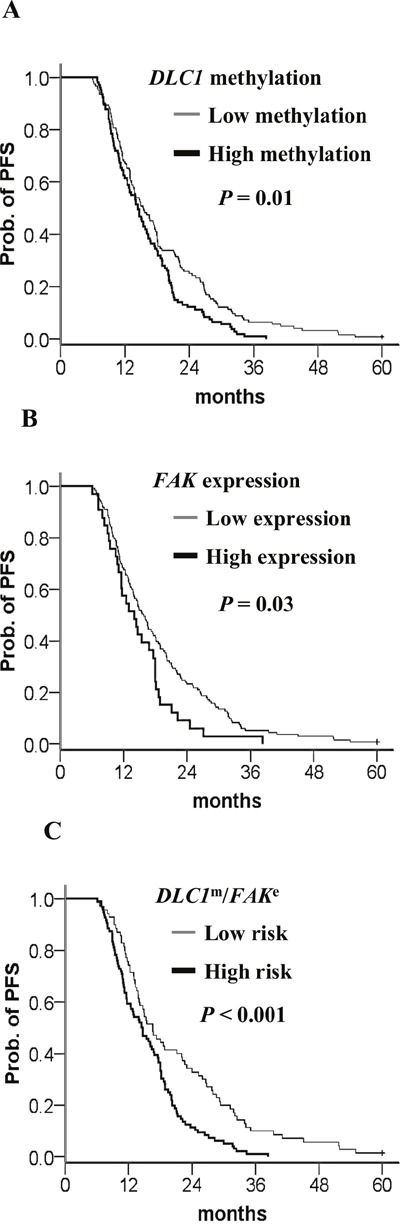
Kaplan–Meier curves of the risk groups in TCGA ovarian cancer patients with suboptimal debulking and the probability of PFS predicted by the *DLC1* methylation status, *FAK* expression status, and combined signatures PFS stratified by **(A)** *DLC1* methylation status. Straight line: low methylation; bold line: high methylation. **(B)** *FAK* expression status. Straight line: low expression; bold line: high methylation. **(C)** *DLC1* methylation and *FAK* expression signature. Straight line: low-risk; bold line: high-risk. Low risk means *DLC1* low methylation and *FAK* low expression. The high-risk group included any combination of *DLC1* methylation with *FAK* expression other than the low-risk group. *P* values were calculated using a log-rank test.

In a Kaplan–Meier survival analysis of patients with suboptimal debulking, high *DLC1* methylation, *FAK* expression, and a high-risk combination of these two significantly decrease PFS (Figure 7A–7C). A Cox proportional hazard model indicated that *DLC1* methylation, *FAK* expression, and the high-risk combination had the notable HRs for PFS (Table 8).

**Table 8:** Univariate and multivariate Cox regression analysis of PFS in TCGA patients with suboptimal debulking ovarian cancer

	Crude HR (95% CI)	*p*	Adjusted HR (95% CI)	*p*
Age	1.01 (0.99, 1.02)	0.24	-	-
Grade	1.21 (0.82, 1.78)	0.34	-	-
FIGO stage III, IV vs II	1.17 (0.60, 2.29)	0.64	-	-
*DLC1* methylation				
High vs low	1.40 (1.07, 1.83)	0.01*	-	-
*FAK* expression				
High vs low	1.55 (1.05, 2.28)	0.03*	-	-
*DLC1*^m^ and *FAK*^e^				
High risk vs low risk^a^	1.79 (1.29, 2.49)	< 0.001*	-	-

HR: hazard ratio; CI: confidence interval. ^a^Low risk means *DLC1* low methylation and *FAK* low expression. The high-risk group included any combination of *DLC1* methylation with *FAK* expression other than the low-risk group. Low risk means *DLC1* low methylation and *FAK* low expression. *p < 0.05.

## DISCUSSION

In the present study, we used a genome-wide approach through the integration of methylomics and genomics analyses to discover genes that are involved in the adhesion of ovarian cancer. Using this approach, we identified four highly methylated genes (*CD97*, *CTNNA1*, *DLC1*, *HAPLN2*) and three genes (*LAMA4*, *LPP*, *MFAP4*) with low methylation that were associated with poor PFS. Furthermore, our results focused on methylation status of CAMs and revealed a 3-gene (*CTNNA1*, *DLC1*, *MFAP4*) methylation signature with a prognostic significance that may provide a new biomarker for personalized treatment in ovarian cancer.

In this era of personalized medicine, we can stratify patients according to their pharmacogenomics and individual genetic differences that determine the response to chemotherapeutics [37]. Furthermore, we may provide mechanism-based and biomarker-driven therapeutics to improve treatment outcome. DNA hypomethylating agents can restore the expression of epigenetically silenced tumor suppressor genes and result in antitumor activity [38]. Decitabine restores the sensitivity toward carboplatin in patients with platinum-resistant ovarian cancer [39]. However, DNA hypomethylating agents lack specificity and may lead to undesirable effects such as re-expression of oncogenes [40]. Application of demethylation therapies in patients with ovarian cancer carrying a specific methylation signature requires clinical trials to confirm their efficacy. CAMs involved in the process of cancer progression have been identified. Monoclonal antibodies targeting CAMs such as integrin and EpCAM have been developed, but the results have been unsatisfactory. Combinations of therapies targeting different CAMs are likely to be more effective and should be explored for their future clinical application. Ovarian cancer patients with suboptimal debulking have an inferior response to adjuvant chemotherapy, PFS, OS, and they used to be regarded as a homogenous group. In the present study, we found high *DLC1* methylation was an independent risk factor for poor PFS. Furthermore, *FAK* expression further increased the hazard ratio for PFS in patients with suboptimal debulking. Small molecule FAK inhibitors showed promising clinical activity to decrease tumor growth and metastasis in preclinical models. Clinical trials are ongoing [33]. FAK inhibitors may be used to improve outcomes in patients with suboptimal debulking who have high *DLC1* methylation and high *FAK* expression.

**Figure 8:**
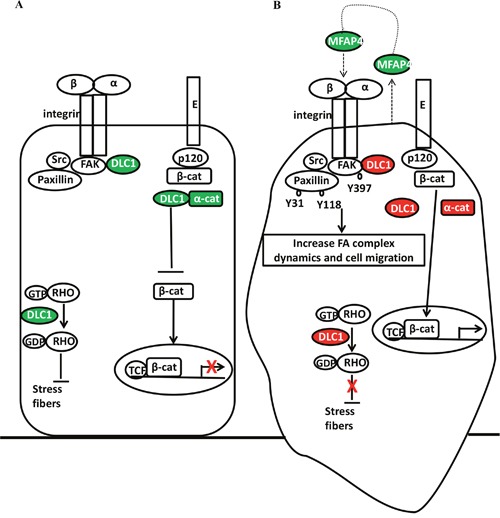
Adhesion molecules (DLC1, α-catenin, MFAP4) interacted with focal adhesion complex, adherens junction, and Rho GTPase Adhesion-related genes that are expressed or low methylation overexpressed (green) and high methylation silenced (red) correlated with focal adhesion complex, adherens junction, and Rho GTPase in ovarian cancer **(A)** without or **(B)** with epigenetic adhesion signatures. E: E-cadherin; p120: p120 catenin; α-cat: α-catenin; β-cat: β-catenin; GTP: guanosine triphosphate; GDP: guanosine diphosphate; TCF: transcription factor; FAK Y397: FAK Y397 phosphorylation; Paxillin Y118 and Y31: Paxillin Y118 and Y31 phosphorylation; FA: focal adhesion.

In the 3-gene methylation signature, high methylation of *CTNNA1* and *DLC1* are associated with a poor prognosis. Downregulation or loss of human α-catenin gene (*CTNNA1*) expression is seen in many cancer cell lines and primary cancer tissues, and α-catenin is recognized as a putative tumor suppressor [41]. Reduced expression of α-catenin is proposed as an important step in ovarian tumorigenesis [42]. α-catenin is involved in the regulation of β-catenin in the Wnt/β-catenin pathway [41]. *DLC1* is a tumor suppressor and its expression is lost or downregulated in multiple common cancers [43]. DLC1 is a Rho GTPase-activating protein (GAP) and mainly regulates Cdc42 [44]. Focal adhesion allows the cell to attach to the extracellular matrix (ECM) through the interaction of the integrins with their extracellular ligands, and intracellular assemblies of multiple proteins which link to the actin cytoskeleton [45]. DLC1 is involved in the process of focal adhesion and stress fiber formation [44]. Expression of DLC1 has a negative correlation with expression of pFAK Y397 in advanced ovarian cancer [36]. Integrin engagement with ECM can activate FAK through autophosphorylation at the Y397 site, which may, in turn, lead to paxillin phosphorylation and affect focal adhesion dynamics [46, 47]. DLC1 competes with paxillin to bind FAK and reduce paxillin phosphorylation [48]. DLC1 acts with FAK to downregulate paxillin turnover independent of its GAP activity [49]. Moreover, DLC1 interacts with α-catenin to stabilize adherens junction and suppress RhoA, RhoC GTPase activities to induce E-cadherin expression through the Rho GAP function [50, 51]. In our proposed model, high methylation of *DLC1* could lead to loss of CDC42, Rho GTPase regulation, and loss of FAK and paxillin phosphorylation inhibition while the integrin receptor is activated. Additional high methylation of *CTNNA1* may lead to instability of the adherens junction, which may increase cytosol levels of β-catenin and increase the chance of nuclear translocation (Figure 8B).

Microfibrillar-associated protein 4 (MFAP4) is an ECM glycoprotein that binds to collagen and elastin [52]. It has a fibrinogen-like domain in the C-terminal region and an Arg-Gly-Asp (RGD) sequence in the N-terminal region that can be a binding motif for cellular surface integrins, which may involve in cell adhesive activity [53, 54]. The role of MFAP4 in cancer is not clear. MFAP5 (which was previously called: MAGP2, microfibril-associated glycoprotein 2) is a secretory protein which prolongs ovarian cancer cell survival, and increases endothelial cell motility, survival through the αvβ3 integrin receptor. MFAP5 is a poor prognostic factor in ovarian cancer and its increased expression is correlated with microvessel density [55]. MFAP4 promotes vascular smooth muscle cell proliferation and migration via the same integrin receptor [56]. Because MFAP4 and MFAP5 are ECM proteins and both act through the αvβ3 integrin receptor, we speculate that MFAP4 has a similar biological function to MFAP5. Low methylation of *MFAP4* may increase secretory MFAP4, which may act as an autocrine agent by binding to the cancer cell integrin receptors, and as a paracrine agent by binding to endothelial cell integrin receptors to increase angiogenesis (Figure 8B). Vitronectin (VTN) is also an ECM glycoprotein with an RGD sequence and binds to the integrin αvβ3 receptors, which may promote cell attachment, spread, and migration [57]. Ovarian cancer cells synthesize VTN and use VTN to organize the adhesion [58]. In the present study, low methylation of VTN is significantly associated with poor OS. VTN has been reported to facilitate disease progression in breast cancer and melanoma [59, 60]. Therefore, VTN could be an important prognostic factor and a therapeutic target.

## CONCLUSION

We identified novel methylated adhesion-related genes that can predict PFS in ovarian cancer by integration of methylomics and genomics analyses. We proposed a methylation gene signature with *CTNNA1*, *DCL1*, and *MFAP4*, which can further identify those patients with a poor prognosis. The potential of this methylation signature was validated in independent datasets. Using this methylation signature can help us to identify those who may have a poor response to current treatment, and delivery of customized epigenetic and targeted therapies might improve the outcome. The detection of tumor-specific methylation in cell-free DNA from serum or plasma is appealing [61]. The clinical application of DNA methylation signature testing using cell-free DNA warrants further investigation.

## MATERIALS AND METHODS

### Methylomics and transcriptomics datasets for biomarker discovery

A methylomics dataset of 391 high-grade serous ovarian cancer samples was constructed using a HumanMethylation27 BeadChip (Illumina, San Diego, CA, USA) [32]. We downloaded level 3 methylation data from TCGA data portal (http://cancergenome.nih.gov/) and used clinical data variables, including age at diagnosis, tumor stage, tumor grade, tumor histology, progression-free status, and vital status. The patient characteristics are listed exhaustively in Table 1. The methylation level was shown as β-values normalized by normal background. The methylation profiling of 27,578 highly informative CpG sites located the promoters of 14,475 genes. We selected 1057 probes (551 genes) that were annotated with adhesion-related function. Next, we selected 914 probes (located at the promoter of 524 genes), which had available values of all samples in the TCGA dataset, which were used for further analysis.

The transcriptomics dataset included 585 data of high-grade serous ovarian cancer samples and eight data of adjacent normal samples and was performed using an Affymetrix HT-HG-U133A GeneChips (Santa Clara, CA, USA). Level 3 data was downloaded from the TCGA data portal. To limit the 524 adhesion-related genes, we integrated transcriptomics data and selected genes, which were calculated using differential gene expression using a *t*-test (*p* < 0.05) and low expression in cancer tissues. Finally, we selected 183 probes (106 genes) to conduct survival analyses.

### Methylomics datasets for validation

The first dataset was generated using ovarian cancer tissues collected from the Tri-Service General Hospital (TSGH). After excluding patients with suboptimal debulking, we selected 37 samples of stage I–IV ovarian cancer for epigenomics analyses using an Infinium HumanMethylation27 BeadChip and collected their demographic and clinical data. The study was conducted with approval from the Institutional Review Board of the TSGH.

A second dataset, from the Australian Ovarian Cancer Study (AOCS) database, included 91 high-grade serous tumors. The DNA methylation levels were quantified using an Illumina Human Methylation 450 BeadChip. All clinical parameters and methylation levels were available from the AOCS data portal.

### Survival analysis

For survival analysis, we used the average methylation level of each gene in all samples as the cutoff values in each methylomics dataset. A methylation level, which was higher or lower than the cutoff value indicates high or low methylation. Kaplan–Meier analysis estimates the probability of PFS by using a log-rank test. The univariate and multivariate Cox regression analyses were used to estimate the hazard ratio (HR) and 95% confidence interval (CI) for the risk of clinicopathological characteristics and each candidate gene. Statistical analyses were performed using SPSS for Windows (version 20.0; IBM Corp, Armonk, NY, USA). All analyses were two-sided, and *p* < 0.05 was regarded as significant.

## SUPPLEMENTARY FIGURE



## Abbreviations

CAMs: cell adhesion molecules
ECM: extracellular matrix
TCGA: The Cancer Genome Atlas
PFS: progression-free survival
OS: overall survival
TSGH: Tri-Service General Hospital
AOCS: Australian Ovarian Cancer Study
Ig-CAMs: immunoglobulin-like CAMs
EpCAM: epithelial cell adhesion molecule
CTNNA1: human alpha-catenin
DLC1: human deleted in liver cancer 1
MFAP4: microfibrillar-associated protein 4
FAK: focal adhesion kinase
HR: hazard ratio
CI: confidence interval
RGD: Arg-Gly-Asp
