# Supplementary material for: An epigenetic signature of adhesion molecules predicts poor prognosis of ovarian cancer patients
Source: Oncotarget. 2017 Jun 16;8(32):53432–49. doi: 10.18632/oncotarget.18515 (PMC5581121; doi:10.18632/oncotarget.18515)
Supplement: Supplementary file 1 [file oncotarget-08-53432-s001.pdf]

An epigenetic signature of adhesion molecules predicts poor prognosis of ovarian cancer patients

SUPPLEMENTARY INFORMATION

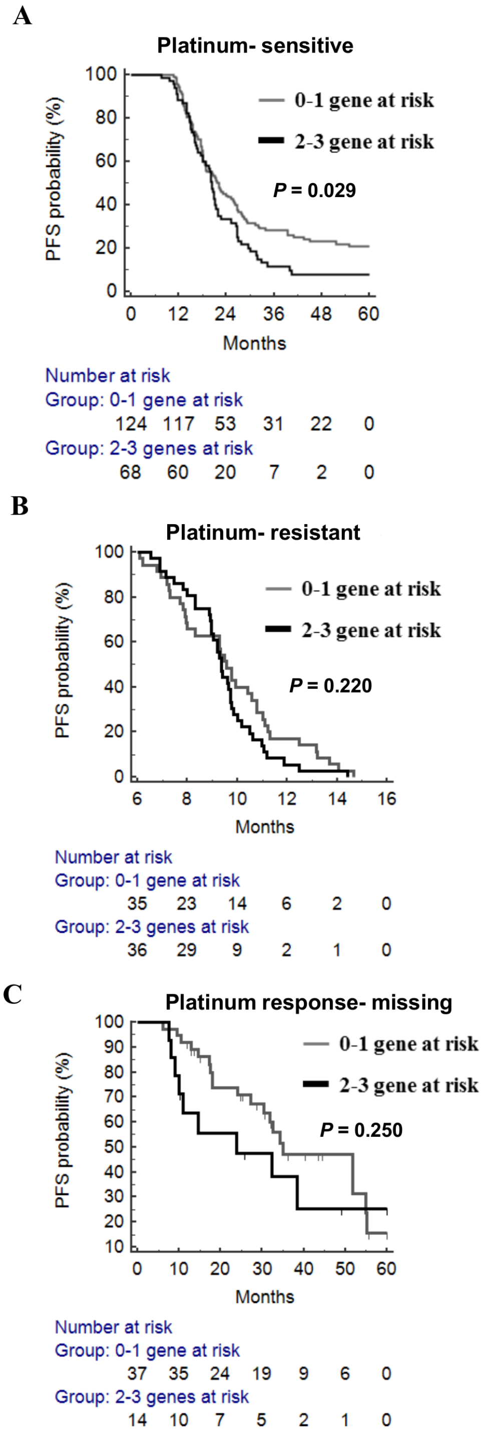

Supplementary Figure 1: The prognostic significance of epigenetic adhesion signatures in patients with differential platinum sensitivity in TCGA patients with high-grade serous ovarian cancers. **(A)** Platinum-sensitive. **(B)** Platinum-resistant. **(C)** Platinum response missing. Gray line: 0–1 risk-related genes; black line: any 2–3 risk-related genes.
